# Correction: Perin et al. Rabies Virus-Neutralizing Antibodies in Free-Ranging Invasive Wild Boars (*Sus scrofa*) from Brazil. *Pathogens* 2024, *13*, 303

**DOI:** 10.3390/pathogens13080643

**Published:** 2024-07-31

**Authors:** Patricia Parreira Perin, Talita Turmina, Carmen Andrea Arias-Pacheco, Jonathan Silvestre Gomes, Lívia de Oliveira Andrade, Natália de Oliveira Zolla, Talita Oliveira Mendonça, Wilson Junior Oliveira, Willian de Oliveira Fahl, Karin Correa Scheffer, Rene dos Santos Cunha Neto, Maria Eduarda Rodrigues Chierato, Enio Mori, Artur Luiz de Almeida Felicio, Guilherme Shin Iwamoto Haga, Maria Carolina Guido, Luiz Henrique Barrochelo, Affonso dos Santos Marcos, Estevam Guilherme Lux Hoppe

**Affiliations:** 1 Department of Pathology, Reproduction and One Health, São Paulo State University, Jaboticabal 14884900, Braziljunior.oliveira@unesp.br (W.J.O.); 2 Laboratory of Rabies Diagnosis, Pasteur Institute, São Paulo 01311090, Brazil; wofahl@pasteur.saude.sp.gov.br (W.d.O.F.); ksferreira@pasteur.saude.sp.gov.br (K.C.S.); mchierato1@gmail.com (M.E.R.C.); enio@usp.br (E.M.); 3 Agricultural Defense Coordination, Department of Agriculture and Supply of the State of São Paulo, Campinas 13070178, Brazilguilherme.haga@sp.gov.br (G.S.I.H.);

There was an error in the original publication [1]. It was stated that two serum samples obtained from wild boars hunted in the municipality of Paraíso tested positive in the RFFIT, instead of one. 

A correction has been made to the first paragraph of Section 3, as follows: 

However, seven serum samples obtained from wild boars hunted in Monte Azul (four samples), Paraíso (one sample), Cajobi (one sample), and Colina (one sample).

In the original publication [1], there was a mistake in Figure 2 as published. On the map, the municipality of Paraíso shows two positive samples instead of one. The corrected Figure 2 appears below. 

The authors state that the scientific conclusions are unaffected. This correction was approved by the Academic Editor. The original publication has also been updated.

## Figures and Tables

**Figure 2:**
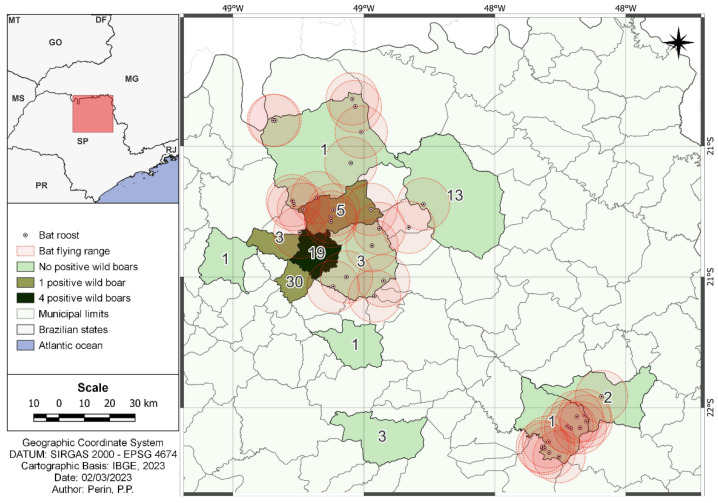
Map highlighting the number of serum samples that tested positive in the RFFIT test, along with the location of bat roosts within a 10 km perimeter of their flying range. The numerical values within each city’s area represent the number of wild boars sampled at each location.
