# Supplementary material for: Rabies Virus-Neutralizing Antibodies in Free-Ranging Invasive Wild Boars (Sus scrofa) from Brazil
Source: Pathogens. 2024 Apr 7;13(4):303. doi: 10.3390/pathogens13040303 (PMC11054148; doi:10.3390/pathogens13040303)
Supplement: Supplementary file 1 [file pathogens-13-00303-s001.zip › pathogens-2920672-supplementary.pdf]

Table S1: Summary of results obtained for wild boar samples in rapid fluorescent foci inhibition test, direct fluorescent antibody test, viral isolation, quantitative reverse transcription polymerase chain reaction, and histopathological techniques, Brazil, 2024.

	**Serum (n=72)**	**Central Nervous System (n=75)**			
**ID**	**RIFFIT (n=72)**	**DFA (n=46)**	**VI (n=46)**	**rt-qPCR (n=24)**	**Histopathology (n=48)**
1	+	np	np	-	np
2	-	np	np	-	np
3	-	np	np	-	np
4	-	np	np	-	np
5	-	np	np	-	np
6	+	np	np	-	np
7	+	np	np	-	np
8	-	np	np	-	np
9	-	np	np	-	np
10	-	-	-	np	np
11	-	np	np	-	np
12	np	np	np	-	np
13	-	np	np	-	np
14	np	np	np	-	np
15	-	np	np	-	np
16	-	np	np	-	np
17	-	-	-	np	np
18	-	np	np	-	np
19	np	np	np	-	np
20	-	np	np	-	np
21	+	np	np	-	np
22	-	np	np	-	np
23	-	np	np	np	np
24	-	np	np	np	np
25	np	np	np	-	np
26	-	np	np	-	np
27	-	np	np	-	np
28	-	np	np	-	np
20	-	np	np	np	np
30	-	-	-	np	np
31	-	np	np	np	np
32	-	np	np	np	np
33	-	np	np	np	np
34	-	np	np	np	np
35	-	-	-	np	-
36	-	-	-	np	-
37	-	-	-	np	-
38	-	-	-	np	-
39	+	-	-	np	-
40	-	-	-	np	-
41	-	-	-	np	-
42	+	-	-	np	-

43	-	-	-	np	-
44	-	-	-	np	-
45	-	-	-	np	-
46	+	-	-	np	-
47	-	-	-	np	-
48	-	-	-	np	-
49	-	-	-	np	-
50	-	-	-	np	-
51	-	-	-	np	-
52	-	-	-	np	-
53	-	-	-	np	-
54	-	-	-	np	-
55	np	np	np	np	-
56	-	np	np	np	-
57	-	-	-	np	-
58	-	-	-	np	-
59	-	-	-	np	-
60	-	-	-	np	-
61	-	-	-	np	-
62	-	-	-	np	-
63	-	-	-	np	-
64	-	-	-	np	-
65	-	-	-	np	-
66	-	-	-	np	-
67	-	-	-	np	-
68	-	-	-	np	-
69	-	-	-	np	-
70	-	-	-	np	-
71	np	np	np	np	-
72	-	np	np	np	-
73	-	-	-	np	-
74	-	-	-	np	-
75	-	-	-	np	-
76	-	-	-	np	-
77	-	-	-	np	-
78	np	np	np	np	-
79	-	-	-	np	-
80	np	-	-	np	-
81	np	-	-	np	-
82	np	-	-	np	-

ID: identification; RIFFIT: rapid fluorescent foci inhibition test; DFA: direct fluorescent antibody test; VI: viral isolation; rt-qPCR: quantitative reverse transcription polymerase chain reaction.

+ positive; - negative; np not performed.
